# Lymphatic lacunae of the human eye conjunctiva embedded within a stroma containing CD34^+^ telocytes

**DOI:** 10.1111/jcmm.15354

**Published:** 2020-06-24

**Authors:** Mihnea I. Nicolescu, Mugurel C. Rusu, Liliana M. Voinea, Alexandra D. Vrapciu, Raluca I. Bâră

**Affiliations:** ^1^ Division of Histology Faculty of Dental Medicine "Carol Davila" University of Medicine and Pharmacy Bucharest Romania; ^2^ Radiobiology Laboratory “Victor Babeș” National Institute of Pathology Bucharest Romania; ^3^ Division of Anatomy Faculty of Dental Medicine “Carol Davila” University of Medicine and Pharmacy Bucharest Romania; ^4^ Department of Ophthalmology Faculty of Medicine "Carol Davila" University of Medicine and Pharmacy Bucharest Romania; ^5^ Department of Ophthalmology Bucharest University Emergency Hospital Bucharest Romania

## Abstract

An accurate identification of telocytes (TCs) was limited because of the heterogeneity of cell types expressing the markers attributed to TCs. Some endothelial lineage cells also could fit within the pattern of TCs. Such endothelial cells could line conjunctival lacunae previously assessed by laser confocal microscopy. We have been suggested that an accurate distinction of TCs from endothelial cells in the human eye conjunctiva could be achieved by use of CD31, CD34 and D2‐40 (podoplanin); and that the conjunctival lacunae are in fact lymphatic. We aimed as testing the hypothesis by an immunohistochemical study on human eye conjunctiva biopsy samples. Samples of human eye conjunctiva from 30 patients were evaluated immunohistochemically by use of the primary antibodies: CD34, D2‐40 and CD31. D2‐40 was equally expressed within epithelia and laminae propria. Basal epithelial cells were D2‐40 positive. Within the stromal compartment, the lymphatic marker D2‐40 labelled several lymphatic vessels. CD31 labelled both vascular and lymphatic endothelial cells within the lamina propria. When capillary lymphatics were tangentially cut, they gave the false appearance of telocytes. Blood endothelial cells expressed CD34, whereas lymphatic endothelial cells did not. Stromal CD34‐expressing cells/telocytes were found building a consistent pan‐stromal network which was equally CD31‐negative and D2‐40‐negative. The conjunctival lymphatic lacunae seem to represent a peculiar anatomic feature of eye conjunctiva. They are embedded within a CD34‐expressing stromal network of TCs. The negative expression of CD31 and D2‐40 should be tested when discriminating CD34‐expressing TCs.

## 1 INTRODUCTION

Human conjunctiva is a mucous membrane extending from the eyelid margin to the corneoscleral limbus^1^ containing an epithelium (with goblet cells, Langerhans’ cells and, occasionally, dendritic melanocytes ^2^) attached to the loose lamina propria.^1^ Many leukocytes are present, mostly T cells and macrophages.^2^

Expression of podoplanin (D2‐40) was used to detect conjunctiva lymphatics in both foetal and adult human eyes.^3^ Studies of conjunctiva by in vivo laser scanning confocal microscopy (LSCM) identified conjunctival lacunae,^4^, ^5^, ^6^ still not yet tested for the immune expression of lymphatic markers in humans.

CD34 is so far one of mostly accepted identifier of telocytes (TCs). On the other hand, CD31 (commonly regarded as endothelial marker), and podoplanin—a marker of lymphatic endothelial cells (LECs)—would not be expressed by TCs. Therefore, this panel of markers is equally suited to detect lymphatics, and also to distinguish them from TCs.^7^ Initial TCs studies failed to use a specific lymphatic marker to discriminate them from LECs, as recently discussed.^8^, ^9^, ^10^, ^11^ Caution should be taken in immunohistochemistry because TCs and endothelial tip cells (guides of angiogenic sprouts) may share a comparable morphology.^12^, ^13^, ^14^

We have been suggested that an accurate distinction of TCs from LECs in the human eye conjunctiva could be achieved by using a three‐marker panel: CD31, CD34 and podoplanin and that the large conjunctival lacunae found by in vivo LSCM studies are in fact lymphatic. We therefore tested the hypothesis by an immunohistochemical study on human eye conjunctiva biopsy samples.

## 2 MATERIAL AND METHODS

The immunohistochemical study was performed retrospectively on archived paraffin‐embedded biopsy samples of human eye conjunctiva (N = thirty cases). Patients’ age ranged from 49 to 58 years. Their written informed consent was obtained, the study was approved (approval 4447/23.01.2019) and conducted in accordance with the general principles of medical research, as stated in the Helsinki Declaration. Tissue samples were processed with an automatic tissue processor (Diapath, Martinengo, BG, Italy) with paraffin embedding. Sections cut manually (3μm) were mounted on SuperFrost® electrostatic slides for immunohistochemistry (ThermoScientific, Menzel‐Gläser, Braunschweig, Germany), after HE‐stain evaluation. Negative controls lacked primary antibodies. Primary antibodies (BiocareMedical, Concord, CA, USA) were as follows: for CD34 (Cat# CM084A,B,C, clone QBEnd/10, 1:50), for CD31 (Cat# CM347A,C, clone BC2, 1:200) and for D2‐40 (Cat# CM266A,B,C, clone D2‐40, 1:100). Tissues were deparaffinized and rehydrated; then endogenous peroxidase was blocked using Peroxidazed 1 (BiocareMedical) for 5 minutes. For heat‐induced epitope retrieval, we used Decloaking Chamber (BiocareMedical) and retrieval solution pH 6 (BiocareMedical). Primary antibodies incubation time was 30 minutes (for CD31 and CD34) and 60 minutes (for D2‐40). We used HRP‐based detection systems from BiocareMedical: 4Plus for CD34, MACH4^TM^ for D2‐40 and MACH2^TM^ for CD31, following the producer's instructions. An HRP‐compatible chromogen (DAB) was applied. Sections were counterstained with haematoxylin and rinsed with deionized water. We washed using pH 7.6 TBS solution. Microscopic slides were analysed, and micrographs were acquired using a calibrated Zeiss working station as described previously,^11^ with an AxioImager M1 microscope, an AxioCam HRc camera and AxioVision software (Carl Zeiss, Oberkochen, Germany).

## 3 RESULTS

**Figure 1:**
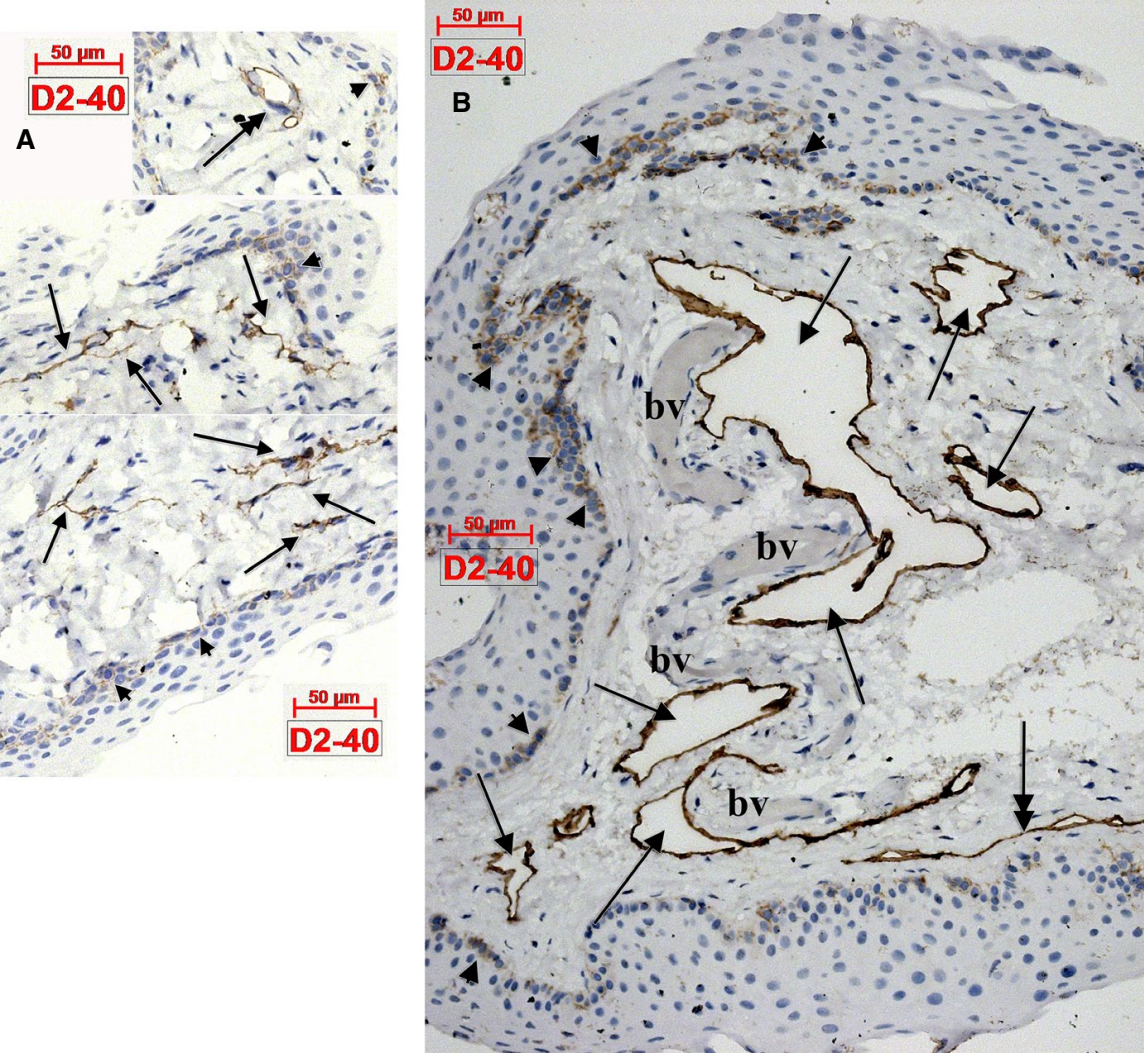
Human eye conjunctiva. (A) Delicate networks of lymphatic capillaries express D2‐40 (arrows) and could easily be confused on slides with telocytes. (B) Expression of D2‐40 is found in basal epithelial cells (arrowheads), as well in the endothelial walls of the lymphatic lacunae (arrows) which border unlabelled blood vessels (bv). Lymphatic collectors (double‐headed arrows in A,B) might be confused with segments of telocytes, especially when sectioned tangentially (B). Occasionally presence of pericytes around these lymphatics (A) might help in differential diagnosis

**Figure 2:**
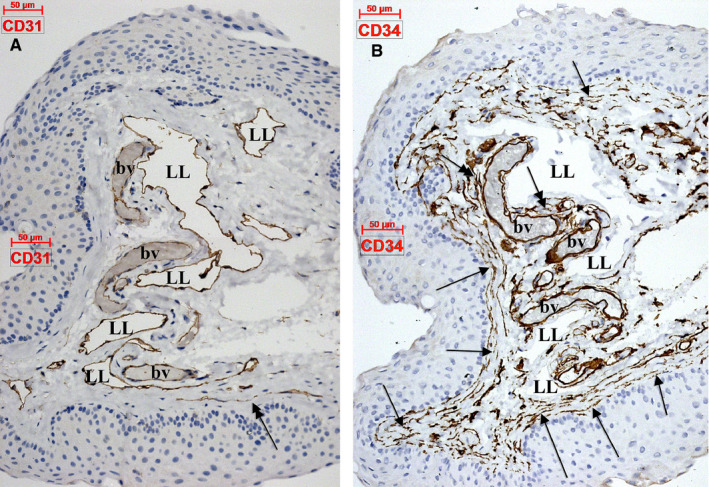
Human eye conjunctiva. (A) CD31 expression in endothelial cells of blood vessels (bv) and lymphatic lacunae (LL). When confirmed lymphatic vessels (D2‐40 positive) are tangentially cut, CD31‐expressing false telocytes may appear on sections (double‐headed arrow). (B) CD34 is expressed in blood vessel (bv) endothelial cells, whereas lymphatic lacunae (LL) are negative. An extensive network of CD34‐expressing stromal cells/telocytes is present in the perivascular (double‐headed arrows) and subepithelial (arrows) lamina propria

D2‐40 was expressed within epithelia and laminae propria. Basal epithelial cells were D2‐40^+^. Within the stromal compartment, the lymphatic marker D2‐40 labelled several lymphatic vessels (Figure 1). Interestingly, we found within the lamina propria also collagen‐embedded large lymphatic lacunae (Figure 1) neighbouring blood vessels and nerves. On successive slides, we adequately distinguished lymphatics from other stromal contents. Thus, CD31 labelled both endothelia and LECs within the lamina propria (Figure 2A). We found CD31‐expressing blood cells (Figure 2A) within microvessels. Tangentially cut lymphatic capillaries may mimic TCs aspect (Figures 1,2A). Blood endothelial cells expressed CD34, whereas LECs did not (Figure 2B). Stromal CD34^+^ TCs were identified building a consistent pan‐stromal CD31^‐^ D2‐40^‐^ network (Figure 2B).

## 4 DISCUSSION

Epithelial expression of podoplanin could relate to the proliferative potential of basal cells, as previously documented.^15^ Podoplanin expression in basal epithelia is high during wound healing^3^, ^16^ and inflammatory responses.^15^, ^17^ Therefore, tissues constantly exposed to exogenous agents and involved in inflammatory processes could display a podoplanin immunoreactivity of the basal epithelium.

We also showed that intrinsic lymphatic vasculature of conjunctiva consists not only from well‐known lymphatic capillaries and collectors,^18^, ^19^ but also from peculiar lymphatic lacunae. Such lacunae described as ‘dilated lymphatic spaces lined with a simple layer of LECs’ were reported in the uterine tube and showed a D2‐40^+^ CD34^+^ expression.^20^ In conjunctiva, the lymphatic lacunae were D2‐40^+^ CD31^+^ CD34^‐^. Nevertheless, caution should be taken when observing slides in light microscopy, as narrow tangentially or longitudinally cut lymphatics could be borders of such lymphatic lacunae.

To the best of our knowledge, podoplanin was not used previously to discriminate conjunctival lymphatic lacunae, in the sense defined by Varga in the uterine tube.^20^ However, in vivo studies of conjunctiva which used LSCM found conjunctival lacunae,^4^, ^5^, ^6^ without discriminating them from lymphatics, which is indeed a limitation of the method. Authors’ descriptions are convergent and fit with lymph‐filled structures: ‘dark amorphous lacunae’,^4^, ^6^ tissue edema seen as ‘multiple black empty spaces’,^21^ ‘wide fluid‐filled hyporeflective microcysts’^22^ or ‘wide intercellular spaces’.^23^ Dye injections demonstrated that the pericorneal lymphatic ring opens into conjunctival lymphatic lacunae (lakes) which, in turn, form superficial and deep networks.^24^ In a different study, these lacunae were indicated as ‘lymphatic bulbs’.^19^

Telocytes are stromal cells mostly identified on two‐dimensional sections as delicate structures, with thin and long prolongations consisting of alternation of dilations (podoms) and thin segments (podomers).^10^, ^25^ They were renamed in 2010 from ‘interstitial Cajal‐like cells’ by Popescu and Faussone‐Pellegrini.^26^ Various studies regarding TCs have attempted to identify specific functional characteristics of these cells, but they often have not used unitary scientific methodology, as recently reported.^9^, ^11^, ^14^, ^27^, ^28^, ^29^, ^30^, ^31^ However, despite the controversies regarding TCs,^9^, ^10^, ^11^, ^32^ they should be included in both the nomenclature and textbooks of histology.^33^ Nevertheless, care should be taken on two‐dimensional slices, as tangentially cut endothelial cells, or lymphatic capillaries, could generate false evidence of TCs.^8^ CD34 is considered in almost all studies identifying TCs as a reliable marker,^9^, ^10^, ^29^ despite the fact that some authors reported as ‘fibrocytes’ either CD34+ stromal cells ^34^ or CD34^+^ Vimentin^+^ myelomonocytic descendants.^35^, ^36^ Indeed, besides the negative expression of CD31 and podoplanin which should be detected in order to accurately identify TCs, there is still at least one different marker to be identified to help discriminating TCs from cells of the hematopoietic lineage. Caution should be taken when CD34 is used alone to discriminate TCs.

## 5 CONCLUSIONS

Conjunctival lymphatic lacunae seem to represent a peculiar anatomic feature of eye conjunctiva. They are embedded within a CD34^+^ stromal network of TCs. Use of CD31, CD34 and podoplanin could discriminate TCs from cells of the endothelial lineages.

## CONFLICTS OF INTERESTS

The authors confirm that there are no conflicts of interest.

## AUTHOR CONTRIBUTION

**Mihnea Ioan NICOLESCU**: involved in conceptualization, investigation and writing‐review and editing. **Mugurel Constantin RUSU**: involved in methodology, investigation and writing‐original draft preparation. **Liliana Mary VOINEA**: involved in investigation, resources and writing‐original draft preparation. **Alexandra Diana VRAPCIU:** involved in investigation, resources and writing‐original draft preparation. **Raluca Iustina BÂRĂ:** involved in investigation, resources and writing‐original draft preparation.

## Data Availability

The data that support the findings of this study are available from the corresponding author upon reasonable request.
